# iPSC‐derived cells stimulate ABCG2 ^+^/NES ^+^ endogenous trabecular meshwork cell proliferation and tissue regeneration

**DOI:** 10.1111/cpr.13611

**Published:** 2024-02-14

**Authors:** Gaiping Xi, Pengchao Feng, Xiaoyan Zhang, Shen Wu, Jingxue Zhang, Xiangji Wang, Ailing Xiang, Wenhua Xu, Ningli Wang, Wei Zhu

**Affiliations:** ^1^ Department of Pharmacology, School of Pharmacy Qingdao University Qingdao China; ^2^ Beijing Institute of Ophthalmology Beijing Tongren Eye Center, Beijing Tongren Hospital, Capital Medical University, Beijing Ophthalmology & Visual Sciences Key Laboratory Beijing China; ^3^ Beijing Institute of Brain Disorders, Collaborative Innovation Center for Brain Disorders Capital Medical University Beijing China; ^4^ Beijing Key Laboratory of Fundamental Research on Biomechanics in Clinical Application Capital Medical University Beijing China; ^5^ Qingdao Xikai Biotechnology Co., Ltd Qingdao China; ^6^ Department of Inspection Qingdao University Qingdao China; ^7^ Advanced Innovation Center for Big Data‐Based Precision Medicine Beijing University of Aeronautics and Astronautics‐Capital Medical University Beijing China

## Abstract

A major risk factor for glaucoma, the first leading cause of irreversible blindness worldwide, is the decellularisation of the trabecular meshwork (TM) in the conventional outflow pathway. Stem cell‐based therapy, particularly the utilisation of induced pluripotent stem cells (iPSCs), presents an enticing potential for tissue regeneration and intraocular pressure (IOP) maintenance in glaucoma. We have previously observed that differentiated iPSCs can stimulate endogenous cell proliferation in the TM, a pivotal factor in TM regeneration and aqueous humour outflow restoration. In this study, we investigated the response of TM cells in vivo after interacting with iPSC‐derived cells and identified two subpopulations responsible for this relatively long‐term tissue regeneration: ATP Binding Cassette Subfamily G Member 2 (ABCG2)‐positive cells and Nestin (NES)‐positive cells. We further uncovered that alterations of these responsive cells are linked to ageing and different glaucoma etiologies, suggesting that ABCG2^+^ subpopulation decellularization could serve as a potential risk factor for TM decellularization in glaucoma. Taken together, our findings illustrated the proliferative subpopulations in the conventional outflow pathway when stimulated with iPSC‐derived cells and defined them as TM precursors, which may be applied to develop novel therapeutic approaches for glaucoma.

## Graphical abstract

Our findings highlight the potential of iPSC‐based therapy to activate the proliferative capacity of two endogenous subpopulations, ABCG2^+^ cells and NES^+^ cells, holding promise for tissue regeneration in the conventional outflow pathway (Created with BioRender.com).

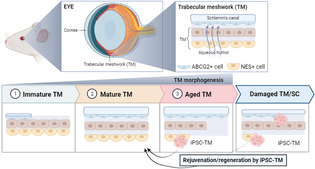

## 1 INTRODUCTION

The trabecular meshwork (TM) and Schlemm's canal (SC) are critical tissues controlling the conventional aqueous humour (AH) drainage from the eye.^1^ Abnormalities in these structures have been linked to the pathogenesis of glaucoma and are considered the primary cause of irreversible blindness worldwide.^2^, ^3^, ^4^, ^5^, ^6^ Recent evidence has increasingly revealled that pharmacological interventions and gene/stem cell‐based therapies targeting the TM and SC can efficiently enhance AH outflow and maintain the eye's intraocular pressure (IOP) homeostasis.^7^, ^8^, ^9^, ^10^, ^11^

Given eye morphogenesis, neural crest cells (NCCs) contribute to TM and SC formations, retaining their multipotency even after migrating into the anterior segment. The persistence of NCCs into adulthood has emerged as a valuable resource for tissue engineering and repair.^12^, ^13^, ^14^, ^15^, ^16^ Over the past 50 years, studies have demonstrated that cells in the Schwalbe line exhibit positivity for certain stem cell biomarkers and continue to actively regenerate damaged TM tissues.^17^, ^18^, ^19^, ^20^ Moreover, our previous studies have revealled that endogenous cells can transit from a quiescent state to an active status immediately after interacting with induced pluripotent stem cell‐derived TM (iPSC‐TM) cells. This initial activation at the time of transplantation enables the TM to regenerate efficiently for an extended period, even though iPSC‐TM cells diminished after transplantation.^7^, ^21^, ^22^, ^23^, ^24^, ^25^ Thus, it is of particular interest to investigate the features of these highly responsive cells. Nevertheless, our understanding of these cells remains limited.

In this study, we investigated these proliferative cells post iPSC‐TM transplantation through immunohistochemistry using specific biomarkers for distinct subpopulations, such as sex‐determining region Y‐box 2 (SOX2) for stem cells, ATP Binding Cassette Subfamily G Member 2 (ABCG2) and Nestin (NES) for NCC and aquaporin 1 (AQP1) for TM. Subsequently, we examined how these cells change in C57BL/6 with age, a glaucoma mouse model (Tg‐MYOC^Y437H^),^26^ cynomolgus macaque after argon laser treatment and human beings with primary open‐angle glaucoma (POAG). Our results not only enhance our understanding of the responsive subpopulation in the conventional outflow pathway after stem cell‐based therapy but also contribute to the discovery of potential treatments for glaucoma.

## 2 MATERIALS AND METHODS

### 2.1 Animals

In total, 2–3 months old C57BL/6 mice were purchased from Beijing Vital River Laboratory Animal Technology Co., Ltd (Beijing, China). Transgenic mice expressing human myocilin^Y437H^ (Tg‐MYOC^Y437H^) were a kind gift from Professor Val C. Sheffield (University of Iowa, USA). Tg‐MYOC^Y437H^ mice under a genetic background of C57BL/6 were used. Mice were housed and bred under standard conditions with a 12/12 h day/night cycle (lights on at 7 am and off at 7 pm), at 23 ± 2°C temperature and 50 ± 5% humidity. All experiments were conducted according to the ARVO Statement for the Use of Animals in Ophthalmic and Vision Research and the laboratory animal care and use guidelines of Qingdao University Medical Center.

### 2.2 Human tissues

Eyes from human donors 1–3 without ophthalmic diseases (Donors 1 and 2: two 4‐year‐old Chinese; Donor 3: 74‐year‐old Caucasian female) or patients with POAG (Donors 4–6: Chinese) were obtained from the First Affiliated Hospital of Harbin Medical University (Donors 1–2; Harbin, China), the Affiliated Hospital of Qingdao University (Donors 4–6; Qingdao, China) and the Lions Eye Bank (Donor 3; Iowa City, IA, USA), respectively. The dissected tissues were used for immunohistochemistry (IHC) analysis. The protocol for human tissue collection was approved by the Ethics Committee of the First Affiliated Hospital of Harbin Medical University and the Affiliated Hospital of Qingdao University and the Eye Bank Association of America in accordance with the tenets of the Declaration of Helsinki.

### 2.3 Cynomolgus macaque tissues

5‐4‐year‐old cynomolgus macaques were provided by JOINN Biologics Co., Ltd (Suzhou, China) and used to create a laser‐induced glaucoma model. As described previously, argon laser photocoagulation (WL: 532 nm; ET: 500 ms; SS: 50 μm; Power: 550–600 mV) was applied unilaterally while the contralateral eye was used as a control. A pneumatonometer (Mentor Classic 30, Reichert Ophthalmic Instruments, Depew, NY) was used for the IOP measurements. Before the measurements, 0.5% proparacaine hydrochloride was applied topically. Eyes displayed an obvious increase in IOP after laser treatment for 2–4 months compared to that of the contralateral eye (IOP in #1: 37.0 vs. 20.0 mmHg; #2: 30.5 vs. 24.0 mmHg; #3: 51.0 vs. 24.0 mmHg, #4: 41.5 vs. 24.5 mmHg) were collected for IHC analysis. All experiments were conducted according to the ARVO Statement for the Use of Animals in Ophthalmic and Vision Research and the laboratory animal care and use guidelines of JOINN Biologics Co., Ltd.

### 2.4 Preparation of mouse iPSC‐TM

As described previously,^22^ the cultured medium conditioned by human trabecular meshwork cells was pooled and sterilised by filtration through mixed cellulose ester membrane filters (0.2 μm pore size; Millipore, Danvers, MA) and applied to induced mouse iPSCs differentiation for 10 days. Differentiated iPSCs, named iPSC‐TM, were separated from undifferentiated cells using a magnetic bead‐based approach^22^ and characterised as TM‐like cells since the expressions of TM biomarkers and dexamethasone‐induced myocilin expression and cross‐linked actin network formation.^7^, ^21^, ^22^, ^23^, ^24^, ^25^ iPSC‐TM generated following this protocol was used in this study.

### 2.5 Intracamerally injection

Mice were subjected to deep anaesthesia using 8% chloral hydrate (0.1 mL/10 g). A total of 50,000 iPSC‐TM cells were resuspended in 3 μL 1 × PBS (Gibco, Grand Island, New York) and injected into the anterior chamber using a 10 μL Hamilton syringe (Boston, MA). Mice having received an equal amount of 1 × PBS (Gibco) were used as the control. Mice were kept on a heated blanket at 37°C until they recovered from the surgery.

### 2.6 Preparation of cryosections

Enucleated mouse, monkey and human eyes were pierced using a 30‐gauge syringe needle (Becton Dickenson, Franklin Lakes, NJ) and fixed immediately by immersion in 4% paraformaldehyde (Thermo, Waltham, MA) for 2 h at room temperature. After rinsing with 1 × PBS (Gibco) and infiltration with 13.3%, 15.0% and 16.7% (wt/vol) of sucrose solutions, the tissues were embedded in Optimal Cutting Temperature compound (OCT; Sakura, Tokyo, Japan) and sectioned to 10 μm thickness on a Leica CM1950 cryostat (Leica, Nussloch, Germany). Cryosectioned tissues were placed on polylysine‐treated slides (Liusheng Co., Ltd, Nantong, China) and stored at −80°C for IHC.

### 2.7 Immunohistochemistry analysis

Cryosectioned tissues were rinsed with 1 × PBS (Gibco) and treated with 0.3% Triton X‐100 (Sigma‐Aldrich, St. Louis, MO) for 5 min. After incubation in the blocking buffer comprised of 1 × PBS (Gibco), 1% BSA (Sigma‐Aldrich) and 0.3% Triton X‐100 (Sigma‐Aldrich) for 1 h at room temperature, samples were incubated with the diluted primary antibodies overnight followed by the corresponding secondary antibodies incubation for 1 h. Cell nuclei were stained with DAPI (Santa Cruz, Dallas, TX). Samples were mounted using an anti‐fluorescence quenching agent (Spark Jade Co., Ltd, Chengdu, China) and imaged by confocal microscopy (Nikon A1MP, Tokyo, Japan; Leica STELLARIS 5, Teaneck, NJ).

The primary antibodies include mouse monoclonal anti‐Aquaporin 1 (Invitrogen, MA5‐25401), rabbit monoclonal anti‐Aquaporin 1 (Abcam, ab168387), mouse monoclonal anti‐RFP (Invitrogen, MA5‐15257), rabbit monoclonal anti‐BCRP/ABCG2 (Abcam, ab229193 and ab207732), Rat monoclonal anti‐ABCG2/CD338 (Novus, NB600‐1079), Chicken polyclonal anti‐Nestin (Novus, NB100‐1604), Rabbit polyclonal anti‐Ki67/MKI67 (Novus, NB500‐170) and Rabbit polyclonal anti‐SOX2 (Abcam, ab97959).

The secondary antibodies were Alexa Fluor® 647 goat anti‐chicken immunoglobulin Y (Abcam, ab150171), Alexa Fluor® 568 goat anti‐rabbit immunoglobulin G (Invitrogen, A11011), Alexa Fluor® 488 goat anti‐mouse immunoglobulin G (Invitrogen, A11001) and Alexa Fluor® 647 goat anti‐rat immunoglobulin G (Invitrogen, A‐21247).

### 2.8 Haematoxylin and Eosin (H&E) staining

Cryosectioned tissues were placed in a glass staining rack and rinsed with water for 15 min. After immersion in the Haematoxylin Solution (G1004, Servicebio Technology Co., Ltd, Wuhan, China) for 5 min, tissues were treated with Haematoxylin Differentiation Solution (G1039, Servicebio Technology), Haematoxylin Bluing Solution (G1040, Servicebio Technology), ascending alcohol solutions (85% and 95%, Sinopharm Chemical Reagent Co., Ltd, Shanghai, China) and EOSIN staining solution (G1001, Servicebio Technology), 100% alcohol solution (Sinopharm Chemical Reagent) and xylene (Sinopharm Chemical Reagent). Samples were mounted using Permount Mounting Solution (Solarbio Co., Ltd, Beijing, China) and imaged by PANNORAMIC MIDI (3DHISTECH Ltd, Hungary).

### 2.9 TM cellularity analysis

To quantify iPSC‐TM cells and endogenous cells in mice high‐magnification images (40×) were used. In each image, cells localised on the TM beams, the region between the pigmented ciliary body and Schlemm's canal and the SC inner wall were counted. Each group contained three to five eyes and four to nine cryosections from each eye were analysed. The average of the counts from four to nine cryosections was taken as the cellularity of each eye. The cellularity of eyes in each group was averaged and used for the following statistical analysis. Quantification was accomplished manually in a double‐blinded fashion. Likewise, endogenous cells in cynomolgus macaque and human beings were quantified at 20× magnification.

### 2.10 Statistical analysis

The Shapiro–Wilk test was used to test for normality. A two‐tailed *t*‐test was applied for statistical analysis in the right panels of Figure 2B,D. One‐way ANOVA was performed for the rest of the statistical analyses. All tests were performed in GraphPad Prism 7.

## 3 RESULTS

### 3.1 Identification of proliferative subpopulations responsible for tissue regeneration

**FIGURE 1:**
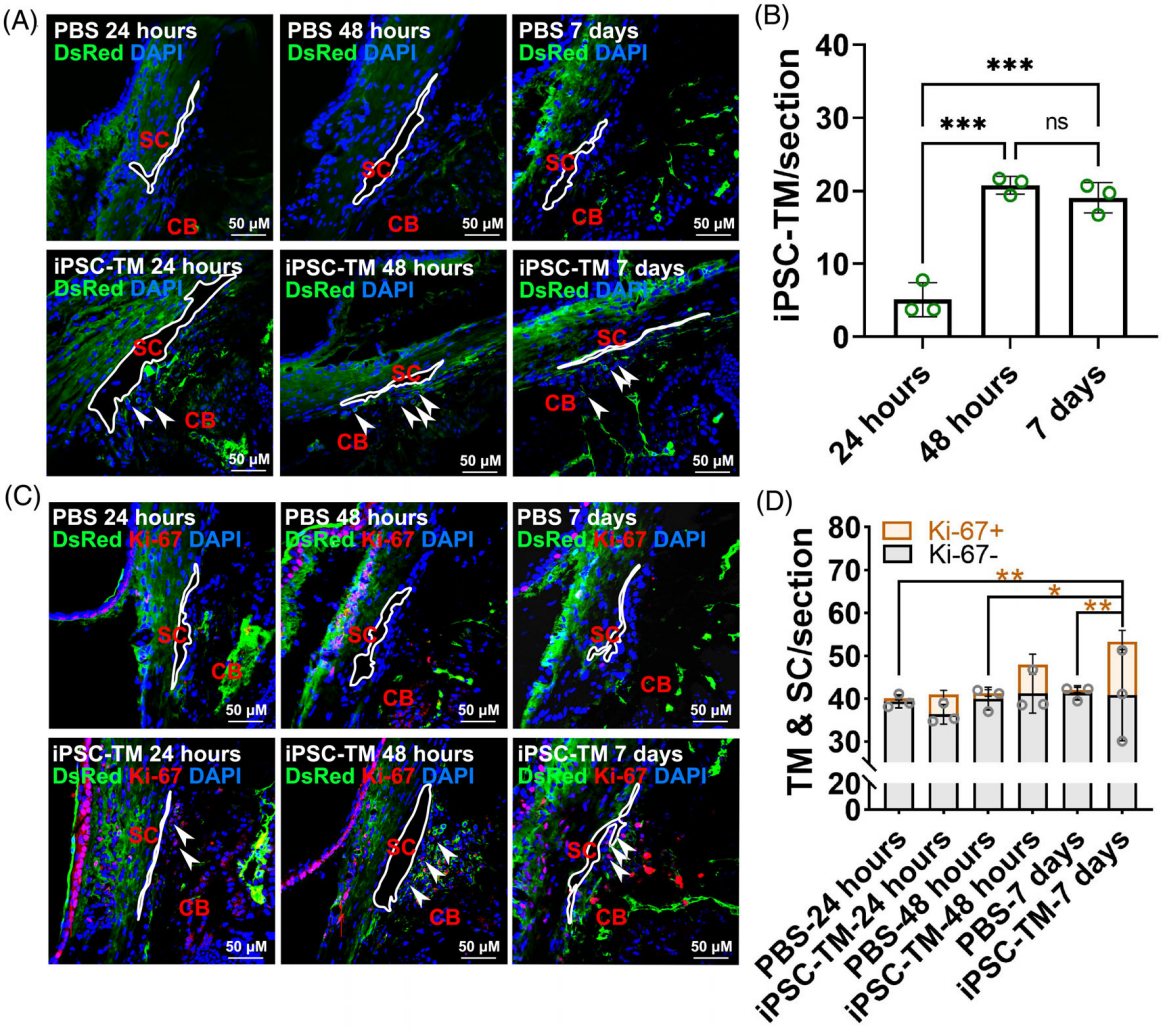
iPSC‐based therapy drives TM regeneration through stimulating endogenous cell proliferation. (A) Immunohistochemical staining of DsRed (green) in PBS and iPSC‐TM recipients after 24 h (*N* = 3), 48 h (*N* = 3), or 7 days (*N* = 3) of transplantation. (B) Quantification of iPSC‐TM cellularity in the conventional outflow region. (C) Immunohistochemical staining of Ki‐67 (red) and DsRed (green) in PBS and iPSC‐TM recipients after 24 h (*N* = 3), 48 h (*N* = 3), or 7 days (*N* = 3) of transplantation. (D) Ki‐67^+^ or Ki‐67^−^ endogenous cells located on the TM beams and Schlemm's canal inner wall are counted. 24 h: 5–6 (PBS) and 4–5 (iPSC‐TM) cryosections/eye, 48 h: 4–6 (PBS) and 5–6 (iPSC‐TM) cryosections/eye, 7 days: 4 (PBS) and 4–7 (iPSC‐TM) cryosections/eye. *p < 0.05, **p < 0.01, ***p < 0.001 by one‐way ANOVA. In panel D, orange stars indicate the statistically significant differences in Ki‐67^+^ cells. Arrowheads indicate DsRed^+^ iPSC‐TM (A) and Ki‐67^+^ endogenous cells (C). White circles indicate Schlemm's canal. Scale bars, 50 μm. CB, Ciliary body; SC, Schlemm's canal.

iPSC‐TM generated following our previous protocol was transplanted into the eyes of 2‐3‐month‐old C57BL/6 mice (*N* = 3 mice). Cells were injected unilaterally (*N* = 3 eyes) and the contralateral eye received the same amount of PBS and was used as a negative control (*N* = 3 eyes). Although the number of transplanted cells gradually decreased after transplantation,^22^ a substantial population of dsRed^+^ iPSC‐TM cells was observed to anchor to the TM region right from the outset of transplantation, with counts of 5.1 cells/section at 24 h, 20.8 cells/section at 48 h and 19.1 cells/section at 7 days (Figure 1A,B). Furthermore, Ki‐67^+^ endogenous cells were immediately detected after iPSC‐TM anchoring, with counts of 4.6 cells/section at 24 h, 6.7 cells/section at 48 h and 12.5 cells/section at 7 days (Figure 1C,D).

**FIGURE 2:**
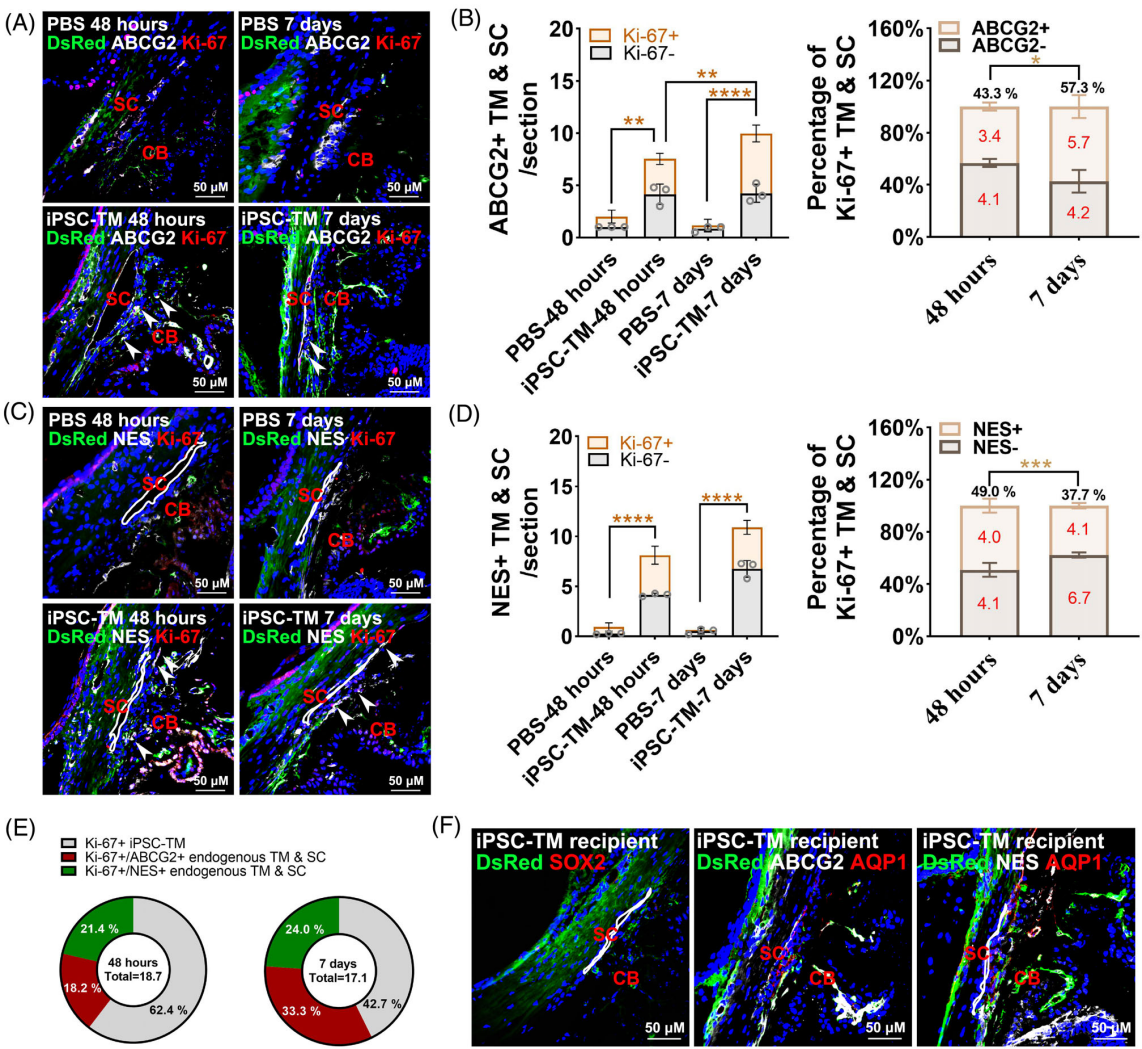
iPSC‐TM stimulates endogenous ABCG2^+^ and NES^+^ cell proliferation. (A) Immunohistochemical staining of ABCG2 (white), Ki‐67 (red) and DsRed (green) in PBS and iPSC‐TM recipients after 48 h (*N* = 3) or 7 days (*N* = 3) of transplantation. (B) Left panel: Quantification of Ki‐67^+^/ABCG2^+^ and Ki‐67^−^/ABCG2^+^ endogenous cells. Right panel: The proportion of Ki‐67^+^/ABCG2^+^ or Ki‐67^−^/ABCG2^+^ endogenous cells relative to ABCG2^+^ endogenous cells. The cell counts for each cluster are shown in each column. (C) Immunohistochemical staining of NES (white), Ki‐67 (red) and DsRed (green) in PBS and iPSC‐TM recipients after 48 h (*N* = 3) or 7 days (*N* = 3) of transplantation. (D) Left panel: Quantification of Ki‐67^+^/NES^+^ or Ki‐67^−^/NES^+^ endogenous cells. Right panel: The proportion of Ki‐67^+^/ NES^+^ or Ki‐67^−^/NES^+^ endogenous cells relative to NES^+^ endogenous cells. The cell counts in each cluster are shown in each column. (E) The proportions of proliferative iPSC‐TM (grey), ABCG2^+^ (red) endogenous cells and NES^+^ (green) endogenous cells in the iPSC‐TM recipients after 48 h (*N* = 3) or 7 days (*N* = 3) of transplantation. Total cell counts are shown in the middle of the Pie Chart. (F) Representative images showing from iPSC‐TM recipients' samples stained with SOX2 (red), ABCG2 (white), NES (white), or AQP1 (red) antibodies. Nuclei were labelled with DAPI (blue). Arrowheads indicate ABCG2^+^/Ki‐67^+^ endogenous cells (A) and NES^+^/Ki‐67^+^ endogenous cells (C). White circles indicate Schlemm's canal. In panels C and F, SC endothelial cells are immunopositive to the ABCG2 antibody. Scale bars, 50 μm. 48 h: 4–8 (PBS) and 4–7 (iPSC‐TM) cryosections/eye, 7 days: 4–6 (PBS) and 4–8 (iPSC‐TM) cryosections/eye. *p < 0.05, **p < 0.01, ***p < 0.001, ****p < 0.0001 by two‐tailed *t*‐tests (for right panels in B and D) or one‐way ANOVA (for the rest). Orange stars indicate the statistically significant differences in ABCG2^+^/Ki‐67^+^ cells and NES^+^/Ki‐67^+^ cells (left panels of B and D). CB, Ciliary body; SC, Schlemm's canal.

To identify which specific group of cells could be stimulated, we stained iPSC‐TM recipients at 48 h and 7 days using ABCG2, NES, SOX2 and AQP1 antibodies as markers for NCCs and adult TM cells. Our IHC results demonstrated that the proliferative ABCG2^+^ cells in the conventional outflow tissue of iPSC‐TM recipients were significantly more abundant than those in eyes receiving PBS (48 h: *p* < 0.01; 7 days: *p* < 0.001; Figure 2A,B). This subpopulation continued to grow over time after transplantation (7 days vs. 48 h: *p* < 0.01; Figure 2B). Quantitative analyses further revealled that Ki67^+^/ABCG2^+^ cells accounted for 43.3% and 57.3% of ABCG2^+^ cells after 48 h and 7 days of transplantation, respectively (Figure 2B).

In comparison to ABCG2^+^ cells, NES^+^ cells displayed remarkably similar behaviours following their interaction with iPSC‐TM (48 h: *p* < 0.0001; 7 days: *p* < 0.0001; Figure 2C,D). The only discernible difference was that the number of NES^+^ cells did not continuously increase after transplantation (Figure 2D). These observations were summarised as a pie chart (Figure 2E).

However, iPSC‐TM recipients didn't show any presence of cells expressing SOX2 (Figure 2F). Additionally, the filtering regions where ABCG2^+^ or NES^+^ cells simultaneously expressing AQP1 were not observed (Figure 2F). Therefore, it is improbable that iPSC‐based tissue regeneration can be attributed to TM stem cells (SOX2^+^ cells) and adult TM cells (AQP1^+^ cells).

### 3.2 Age‐related changes in ABCG2 ^+^/NES ^+^ cells in C57BL/6 mice

**FIGURE 3:**
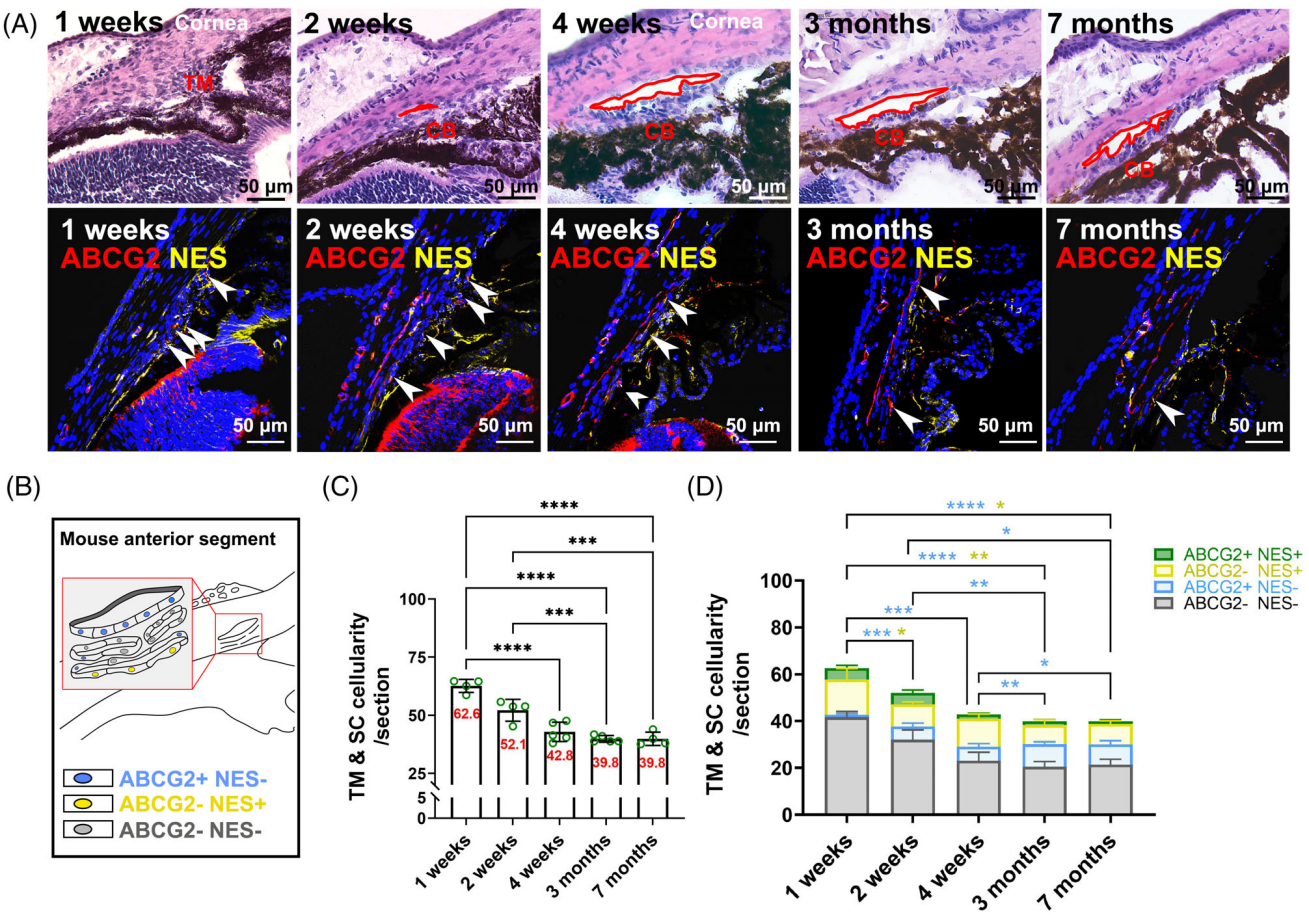
Age‐related changes in ABCG2^+^ and NES^+^ cells in male C57BL/6. (A) Representative images showing the anatomic structures of the anterior segment (Top panels: H&E staining) and the labeling using ABCG2 (red) and NES (yellow) antibodies (Lower panels: IHC staining) in male C57BL/6 at 1 week (*N* = 4, 5–6 cryosections/eye), 2 weeks (*N* = 4, 5–7 cryosections/eye), 4 weeks (*N* = 5, 5–7 cryosections/eye), 3 months (*N* = 5, 4–6 cryosections/eye) and 7 months old (*N* = 4, 5–9 cryosections/eye). Red circles indicate Schlemm's canal. Ciliary body: CB. Nuclei were stained with DAPI (blue). Scale bars, 50 μm. (B) Schematic illustration of the locations of ABCG2^+^/NES^−^ (blue), ABCG2^−^/NES^+^ (yellow) and ABCG2^−^/NES^−^ (grey) cells in mouse iridocorneal angle. (C) Quantification of cells with distinct markers in male C57BL/6 mice, with total cell counts shown in the columns. (D) Quantification of ABCG2^+^/NES^+^ (green), ABCG2^−^/NES^+^ (yellow), ABCG2^+^/NES^−^ (blue) and ABCG2^−^/NES^−^ (grey) cells in male C57BL/6 mice. *p < 0.05, **p < 0.01, ***p < 0.001, ****p < 0.0001 by one‐way ANOVA. Blue and yellow asterisks indicate the statistically significant differences in ABCG2^+^/NES^−^ and ABCG2^−^/NES^+^ cells, respectively.

The impact of age on ABCG2^+^/NES^+^ cells was further investigated. We collected tissue samples from male C57BL/6 at various stages of life and performed histological analyses (Figure 3A). Within the conventional outflow region, we observed minimal overlap between ABCG2^+^ and NES^+^ cells (Figure 3A,B). Consistent with the age‐associated decrease in TM cellularity, ABCG2^−^/NES^+^ and ABCG2^+^/NES^+^ cellularity in the conventional outflow tissues significantly decreased as mice aged. In contrast, the ABCG2^+^/NES^−^ population showed a significant increase in cellularity with age (Figure 3C,D). These age‐associated changes were also observed in female mice (Figure S1A,B).

### 3.3 Changes in ABCG2 ^+^/NES ^+^ cells in Tg‐MYOC^Y437H^ mice

**FIGURE 4:**
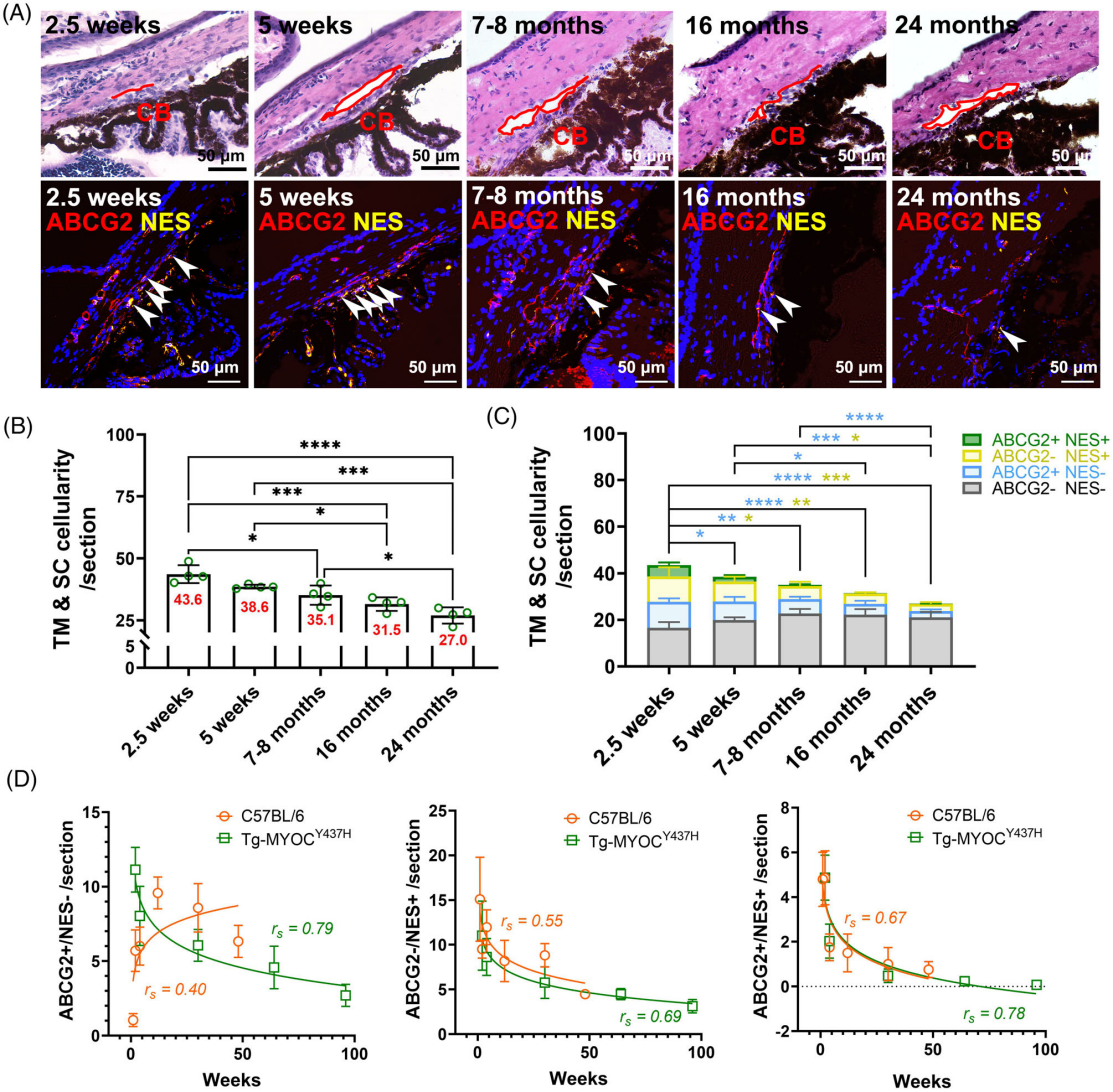
Age‐related changes in ABCG2^+^ and NES^+^ cells in Tg‐MYOC^Y437H^ mice. (A) Left panel: Representative images showing the anatomic structures of the anterior segment (Top panels: H&E staining) and labelling using ABCG2 (red) and NES (yellow) antibodies (Lower panels: IHC staining) in Tg‐MYOC^Y437H^ at 2.5 weeks (*N* = 4, 4–8 cryosections/eye), 5 weeks (*N* = 4, 4–6 cryosections/eye), 7–8 months (*N* = 4, 6–7 cryosections/eye), 16 months (*N* = 4, 4–6 cryosections/eye) and 24 months old (*N* = 4, 6–8 cryosections/eye). Red circles indicate Schlemm's canal. Nuclei were stained with DAPI (blue). Scale bars, 50 μm. (B) Quantification of cells with distinct markers in Tg‐MYOC^Y437H^ mice, with total cell counts shown in the columns. (C) Quantification of ABCG2^+^/NES^+^ (green), ABCG2^−^/NES^+^ (yellow), ABCG2^+^/NES^−^ (blue) and ABCG2^−^/NES^−^ (grey) cells in Tg‐MYOC^Y437H^ mice with blue and yellow asterisks indicating statistically significant differences in ABCG2^+^/NES^−^ and ABCG2^−^/NES^+^ cells, respectively. *p < 0.05, **p < 0.01, ***p < 0.001, ****p < 0.0001 by one‐way ANOVA. (D) Cellularity of ABCG2^+^/NES^−^, ABCG2^−^/NES^+^ and ABCG2^+^/NES^+^ cells cross‐plotted against the age of C57BL/6 (orange) and Tg‐MYOC^Y437H^ (green). Data points are fitted into a semi‐log regression model. CB, Ciliary body.

Tg‐MYOC^Y437H^ mouse is a well‐studied glaucoma model and has been widely applied to enhance our understanding of the critical pathological mechanism in glaucoma, specifically the reduced TM cellularity resulting from severe endoplasmic reticulum stress.^26^ Here, we sought to address whether these proliferative subpopulations could be affected by MYOC^Y437H^. To this end, we quantified the cellularity of ABCG2^+^ and NES^+^ populations in Tg‐MYOC^Y437H^ mice at different ages (Figure 4A,C) and compared the findings to those in C57BL/6 mice (Figure 4D). Our results not only revealled a more severe age‐related decline in TM and SC cellularity but also indicated that the cellularity of all active subpopulations, including ABCG2^+^/NES^−^ cells which increased with age in C57BL/6 mice (Figure 3D), exhibited an age‐associated decrease (Figure 4C,D).

### 3.4 Alterations in ABCG2 ^+^/NES ^+^ cells in monkeys and humans

**FIGURE 5:**
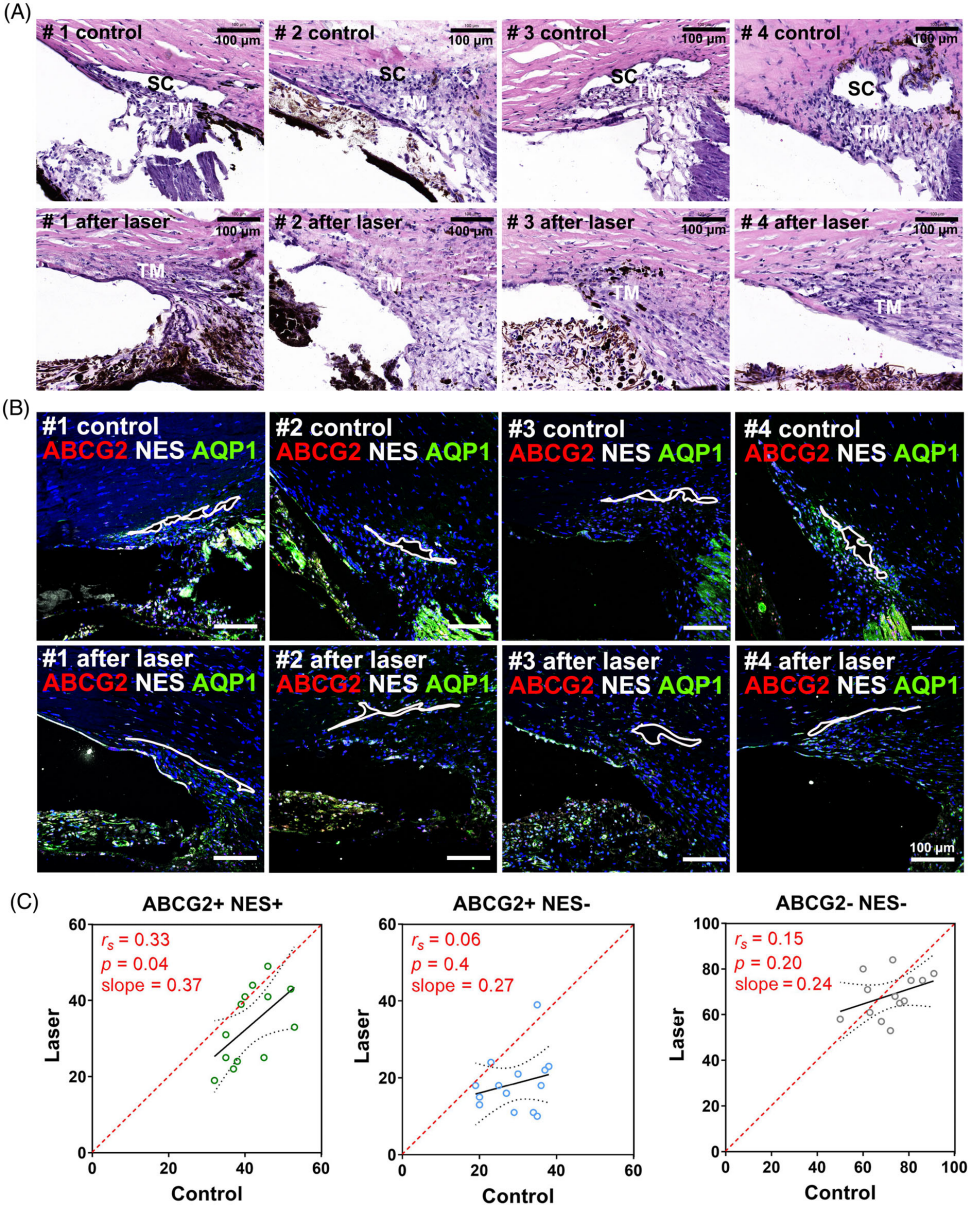
ABCG2^+^ and NES^+^ cells in glaucoma monkeys. (A). Representative images showing the anatomic structures of cynomolgus macaque eyes with (lower panels) and without (top panels) laser treatment. Laser‐induced anatomic abnormalities include accumulated extracellular matrix in the TM, collapsed Schlemm's canal and extensive pigmentation in the TM of the lasered eyes, but not in the contralateral controls. (B) Immunohistochemical staining of ABCG2 (red), NES (white) and AQP1 (green) in the laser‐treated eyes and the contralateral controls. White circles indicate Schlemm's canal. (C) Cellularity of ABCG2^−^/NES^−^ (grey), ABCG2^+^/NES^+^ (green), or ABCG2^+^/NES^−^ (blue) cells in the control eyes cross‐plotted against that in the laser‐treated eyes. *r*_s_, *p* and slope by the Person correlation coefficient are indicated. Data from four pairs of eyes are shown. # 1: 5 (control) and 4 (lasered) cryosections/eye, # 2: 4 (control) and 3 (lasered) cryosections/eye, # 3: 3 (control) and 4 (lasered) cryosections/eye, # 4: 3 (control) and 4 (lasered) cryosections/eye. SC, Schlemm's canal; TM, trabecular meshwork.

We extended our investigation to include anterior segments from *cynomolgus macaques* that had undergone laser treatment and subsequently conducted H&E staining and IHC analyses (Figure 5A,B). Interestingly, we found that monkeys exhibited a higher percentage of ABCG2^+^/NES^+^ cells than other species, with most cells located in the Schwalbe's line (Figures 5B and S2). Moreover, we observed a significant decrease in TM cellularity after argon laser photocoagulation, mainly attributed to the decrease of ABCG2^+^ cells (Figures 5C and S2).

**FIGURE 6:**
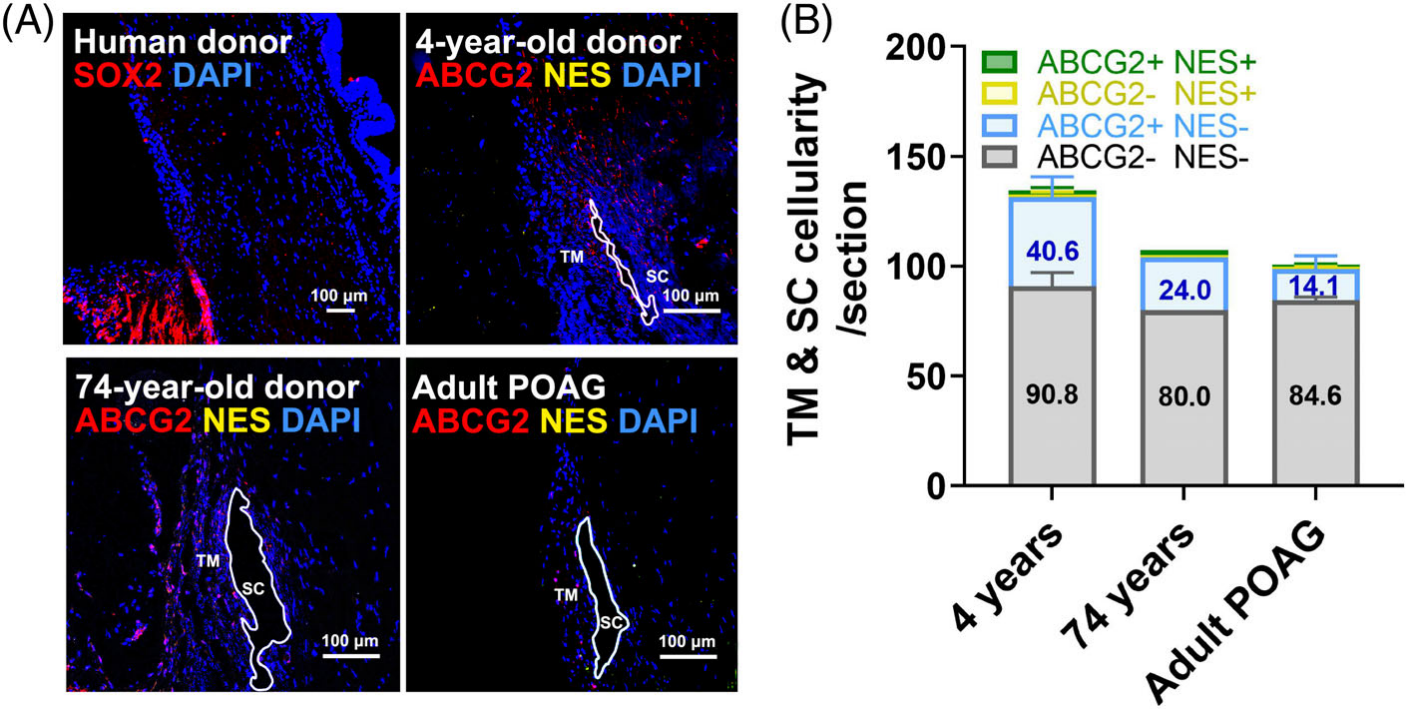
ABCG2^+^ and NES^+^ cells in humans and POAG patients. (A) Immunohistochemical staining of SOX2 (red), ABCG2 (red) and NES (yellow) in the eyes of normal humans (4 years: *N* = 3 eyes, 3–5 cryosections/eye; 74 years: *N* = 1 eye, 3 cryosections/eye) and POAG patients (*N* = 2 eyes, 4–5 cryosections/eye). White circles indicate Schlemm's canal. Scale bars, 100 μm. (B) Quantification of ABCG2^+^/NES^+^ (green), ABCG2^−^/NES^+^ (white), ABCG2^+^/NES^−^ (blue) and ABCG2^−^/NES^−^ (grey) cells in these eyes, with cell count numbers of ABCG2^+^/NES^−^ and ABCG2^−^/NES^−^ subpopulations shown in the columns. SC, Schlemm's canal; TM, trabecular meshwork.

We were unable to detect SOX2‐positive cells in the eyes of normal humans (Figure 6A). Patients afflicted with POAG, the predominant type of glaucoma, showed a reduction in the abundance of ABCG2^+^ cells in the conventional outflow pathway compared to non‐glaucomatous individuals (Figure 6A,B).

## 4 DISCUSSION

Years of dedicated research efforts have culminated in our discovery of a distinct transition zone between the TM and the corneal endothelial layer, hosting a distinct type of NCC progenitor cells expressing many stemness‐related proteins, such as OCT3/4, C‐MYC, ABCG2, NES and SOX9.^27^, ^28^, ^29^ In this study, we found that endogenous cells expressing ABCG2 or NES were significantly activated following interaction with transplanted iPSC‐TM cells, comprising nearly all Ki‐67^+^ endogenous cells (about 92.3% and 94.6% at 48 and 72 h, respectively; Figures 1, 2, 3). However, our IHC results further demonstrated that these proliferative cells did not express SOX2 (Figures 2 and 6). Moreover, unlike the localisation of previously reported NCC progenitors in humans, we observed that ABCG2^+^ cells predominantly aggregated in the inner wall of Schlemm's canal, while NES^+^ cells were primarily situated in the uveal meshwork layers in mice (Figures 3 and 4). These ABCG2^+^ cells and NES^+^ cells only displayed very limited overlap in mice and humans. Therefore, our findings suggest that these proliferative cells are not true NCC progenitors in Schwalbe's line, as reported,^27^ but are more appropriately designated as TM precursors.

Furthermore, we noted that very few cells in Schwalbe's line expressing ABCG2 and NES simultaneously in mice and humans could be indeed the reported NCC progenitors (Figures 2, 3, 4). However, utilising these progenitors for in situ regeneration faces various challenges, primarily stemming from factors in vivo inhibitory factors, such as cell growth inhibitors in aqueous humour and contact inhibition.^27^, ^30^ The proper approach to trigger progenitor proliferation in vivo remains uncertain, but our results suggest a potential avenue wherein transplanted iPSC‐TM may activate these progenitors from a quiescent state, differentiating them into ABCG2^+^ cells and NES^+^ cells. Subsequently, the proliferation capacity of ABCG2^+^ or NES^+^ cells contributes to eventual tissue regeneration. Further studies should be desired to verify this hypothesis. In addition, we also noticed the proportion of ABCG2^+^/NES^+^ cells to be larger in monkeys compared to that in mice and humans, suggesting that the number of progenitors may vary among species.^31^, ^32^

An additional intriguing observation was made when we tracked the trajectory of cell proportions in the above subpopulations with age in mice (Figure 3). We noticed that as NES^+^ cell numbers decreased with age, ABCG2^+^ cell numbers increased in both male and female mice. This observation suggests a potential transition in cell fate from NES^+^ cells to ABCG2^+^ cells during development. Another concept aligns more favourably to explain this observation, which refers to SC development.^33^, ^34^, ^35^, ^36^, ^37^, ^38^ Around postnatal days 10–12 in C57BL/6 mice, the initial formation of Schlemm's canal is marked by the presence of a small endothelial‐lined channel beneath the immature TM beams in the sclera. It is at this developmental stage that ABCG2 may begin to express in Schlemm's canal endothelial cells and correspondingly, we only observed ABCG2^+^ cells in normal C57BL/6 mice after 2 weeks of birth (Figure 3).

We also extended our investigation to Tg‐MYOC^Y437H^ mice, which exhibit similar pathological phenotypes to POAG and explored how these subpopulations change with age. Consistent with our observations in C57BL/6 mice and the previously reported results,^2^, ^5^, ^6^, ^39^ ageing significantly decellularized tissues in the conventional outflow pathway. What's more deleterious, introducing MYOC^Y437H^ accelerated this decellularisation process, especially at a young age (Figure 4B vs. Figure 3C). Moreover, it should be noted that ABCG2^+^/NES^−^ cellularity decreased significantly with age in Tg‐MYOC^Y437H^ mice, which contrasts with the trend observed in C57BL/6 mice (Figure 3). In line with this trend, argon laser treatment, another approach to disrupt the TM^40^ also led to significant decellularization of ABCG2^+^ cells in monkeys (Figure 5). To summarize these results, cell loss in ABCG2^+^ subpopulation may be a major contributor to TM decellularisation in glaucoma (Figure 6). If this is true, it may provide a new strategy for restoring AH outflow in glaucoma.

In summary, our findings highlight the potential of iPSC‐based therapy to activate the proliferative capacity of two endogenous subpopulations, ABCG2^+^ cells and NES^+^ cells, holding promise for tissue regeneration in the conventional outflow pathway.

## AUTHOR CONTRIBUTIONS

Conceptualisation: WZ, NW. Methodology: GX, SW, JZ, XW. Investigation: GX, SW, JZ, XW, AX, FP, ZX. Funding acquisition: WZ, WX. Supervision: WZ. Writing: GX, WZ.

## CONFLICT OF INTEREST STATEMENT

Ailing Xiang is affiliated with Qingdao Xikai Biotechnology Co., Ltd.

## Supporting information


**Figure S1.** Age‐related changes in ABCG2^+^ and NES^+^ cells in female C57BL/6.
**Figure S2.** ABCG2^+^ and NES^+^ cells in glaucoma monkeys.

## Data Availability

The data that support the findings of this study are available from the corresponding author upon reasonable request.
