# Supplementary material for: iPSC‐derived cells stimulate ABCG2 +/NES + endogenous trabecular meshwork cell proliferation and tissue regeneration
Source: Cell Prolif. 2024 Feb 14;57(7):e13611. doi: 10.1111/cpr.13611 (PMC11216930; doi:10.1111/cpr.13611)
Supplement: Supplementary file 1 — Figure S1. Age‐related changes in ABCG2+ and NES+ cells in female C57BL/6. Figure S2. ABCG2+ and NES+ cells in glaucoma monkeys. [file CPR-57-e13611-s001.docx]

**Figure S1**

**
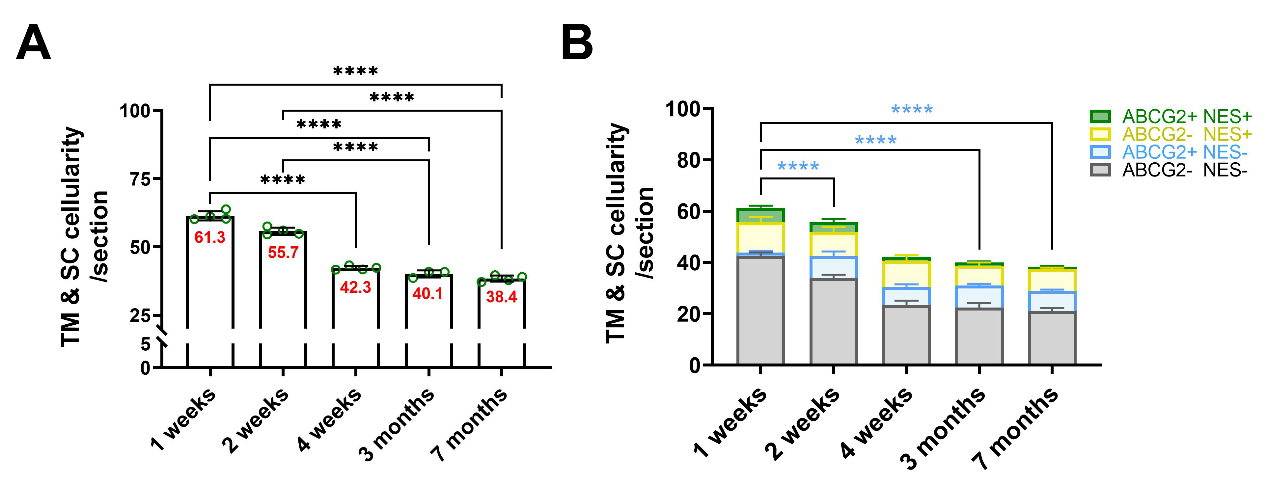
**

**Figure S1. Age-Related changes in ABCG2^+^ and NES^+^ cells in female C57BL/6.** (A). Quantification of four cell subpopulations in female C57BL/6 at 1 week (N = 4, 5-6 cryosections/eye), 2 weeks (N = 4, 5-8 cryosections/eye), 4 weeks (N =4, 4-6 cryosections/eye), 3 months (N = 3, 5-7 cryosections/eye), and 7 months old (N = 4, 5-8 cryosections/eye). (B). Quantification of ABCG2^+^/NES^+^ (green), ABCG2^-^/NES^+^ (yellow), ABCG2^+^/NES^-^ (blue), and ABCG2^-^/NES^-^ (gray) cells in female C57BL/6 mice. **** *P* < 0.0001 by one-way ANOVA. Blue asterisks indicate the statistically significant differences in ABCG2^+^/NES^-^ cells.

**Figure S2**


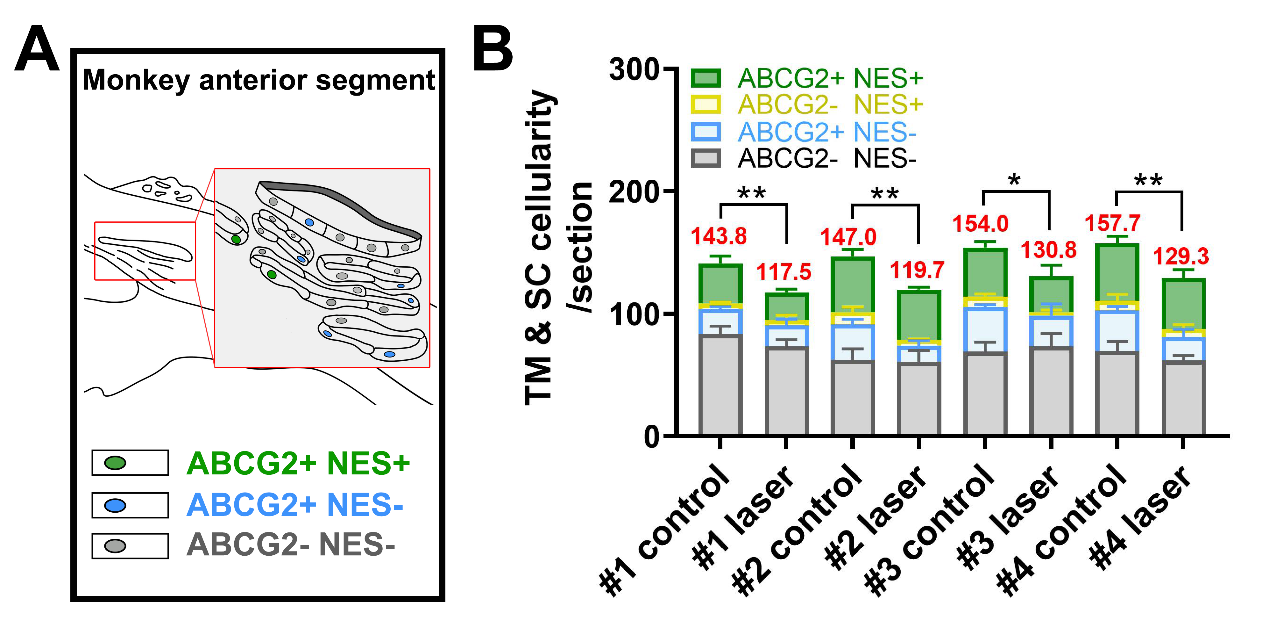


**Figure S2. ABCG2^+^ and NES^+^ cells in glaucoma monkeys. (A).** Schematic illustration of the locations of ABCG2^+^/NES^+^ (green), ABCG2^+^/NES^-^ (blue), and ABCG2^-^/NES^-^ (gray) cells in monkey iridocorneal angle. (B). Quantification of ABCG2^+^/NES^+^ (green), ABCG2^-^/NES^+^ (yellow), ABCG2^+^/NES^-^ (blue), and ABCG2^-^/NES^-^ (gray) cells, with total cell counts shown on the top of the columns. Black asterisks indicate the statistically significant differences between cells in each pair of eyes. * *P* < 0.05, ** *P* < 0.01 by two-tailed t-test.
